# Whole genome sequencing for the investigation of canine mammary tumor inheritance - an initial assessment of high-risk breast cancer genes reveal *BRCA2* and *STK11* variants potentially associated with risk in purebred dogs

**DOI:** 10.1186/s40575-020-00084-w

**Published:** 2020-06-25

**Authors:** Anna L. W. Huskey, Katie Goebel, Carlos Lloveras-Fuentes, Isaac McNeely, Nancy D. Merner

**Affiliations:** 1 Department of Pathobiology, Auburn University College of Veterinary Medicine, Auburn, AL 36849 USA; 2 Department of Drug Discovery and Development, Auburn University Harrison School of Pharmacy, Auburn, AL 36849 USA

**Keywords:** Whole genome sequencing (WGS), Canine mammary tumors (CMT), Inherited risk, Germline mutation, Purebred dogs

## Abstract

**Background:**

Although, in general, cancer is considered a multifactorial disease, clustering of particular cancers in pedigrees suggests a genetic predisposition and could explain why some dog breeds appear to have an increased risk of certain cancers. To our knowledge, there have been no published reports of whole genome sequencing to investigate inherited canine mammary tumor (CMT) risk, and with little known about CMT genetic susceptibility, we carried out whole genome sequencing on 14 purebred dogs diagnosed with mammary tumors from four breed-specific pedigrees. Following sequencing, each dog’s data was processed through a bioinformatics pipeline. This initial report highlights variants in orthologs of human breast cancer susceptibility genes.

**Results:**

The overall whole genome and exome coverage averages were 26.0X and 25.6X, respectively, with 96.1% of the genome and 96.7% of the exome covered at least 10X. Of the average 7.9 million variants per dog, initial analyses involved surveying variants in orthologs of human breast cancer susceptibility genes, *BRCA1*, *BRCA2*, *CDH1*, *PTEN*, *STK11,* and *TP53*, and identified 19 unique coding variants that were validated through PCR and Sanger sequencing. Statistical analyses identified variants in *BRCA2* and *STK11* that appear to be associated with CMT, and breed-specific analyses revealed the breeds at the highest risk. Several additional *BRCA2* variants showed trends toward significance, but have conflicting interpretations of pathogenicity, and correspond to variants of unknown significance in humans, which require further investigation. Variants in other genes were noted but did not appear to be associated with disease.

**Conclusions:**

Whole genome sequencing proves to be an effective method to elucidate risk of CMT. Risk variants in orthologs of human breast cancer susceptibility genes have been identified. Ultimately, these whole genome sequencing efforts have provided a plethora of data that can also be assessed for novel discovery and have the potential to lead to breakthroughs in canine and human research through comparative analyses.

## Plain ENGLISH summary

Despite the advances in sequencing technology and the success of previous canine whole genome sequencing research, we know of no other publications that report using whole genome sequencing to investigate a genetic risk (aka. a risk that can be passed down through generations) for canine mammary tumors in purebred dogs. This canine cancer type is comparable to human breast cancer, and as a result, genes that are known to influence inherited risk for breast cancer were investigated to determine if those same genes played a role in risk for dogs. We whole genome sequenced 14 purebred dogs from four different breeds; each of the dogs within a breed had been tied back to the same family tree (pedigree). From this study, we have identified mutations in genes *BRCA2* and *STK11* that could increase risk for those dogs with the mutations. These mutations seem to be present in some breeds more than others, thus affecting risk differently. Furthermore, the large dataset from this research allows for further exploration to find additional mutations that influence their risk for developing canine mammary tumors.

## Background

The practice of breeding dogs for specific characteristics and traits has resulted in over 190 phenotypically diverse breeds, according to the American Kennel Club [1]. Defined as selective breeding, this practice has cultivated breed-specific gene pools that not only contribute towards each breed’s defining features but also disease susceptibility. To date, over 450 canine genetic diseases have been reported, many of which are monogenic and limited to a specific dog breed(s) [2–4]. Efforts to understand the genetic causes of such diseases began in the 1980s with the first canine genetic mutation identified in 1989 for hemophilia B, an X-linked disorder [5]. Since then, investigating hereditary diseases that segregate in purebred lines/pedigrees have fostered numerous genetic discoveries; over 130 canine hereditary diseases are now genetically explained [2–4]. Through these discoveries, it has been determined that there is much genetic overlap between canine and human disease. Importantly, the elucidation of certain hereditary canine diseases has even led to breakthroughs in human medicine, with diseases such as sleep disorders, Birt-Hogg-Dubé syndrome, and more [3, 6–9].

Interestingly, despite the fact that some dog breeds appear to have an increased risk of certain cancer types, little is known about the etiology. Although, in general, cancer is considered a multifactorial disease, clustering of particular cancers in pedigrees suggests a genetic predisposition [10]. In humans, the study of cancer families has revealed genetic mutations that severely increase lifetime risk of developing particular cancers; for instance, high-risk mutations in *BRCA1*, *BRCA2*, *CDH1*, *PTEN*, *STK11,* and *TP53* all result in hereditary cancer syndromes (such as hereditary breast cancer syndrome, Li Fraumeni syndrome and Cowden Syndrome) associated with an increased risk of breast cancer as well as other cancer types [11]. Therefore, breed or kennel/pedigree-based studies should be a beneficial approach to determine cancer genetic risk in dogs. This approach was successful in identifying the susceptibility locus for multifocal renal cystadenocarcinoma and nodular dermatofibrosis (RCND) in a German Shepherd pedigree [9]. RCND, an inherited cancer syndrome, is an autosomal dominantly inherited trait that is caused by a mutation in the *Birt-Hogg-Dubé* (*BHD*) gene, which is named after the equivalent human cancer syndrome [12–14]. Similar to how the *BHD* mutation in German Shepherds predisposes them to RCND, there are likely yet-to-be-discovered mutations that explain particular cancer incidences in other breeds.

With little known about canine mammary tumor (CMT) genetic susceptibility [15], we decided to carry out whole genome sequencing (WGS) on 14 purebred dogs diagnosed with CMT from four different breeds (Golden Retriever, Siberian Husky, Dalmatian, and Standard Schnauzer). The CMT-affected dogs from each breed were linked back to a common ancestor through pedigree analysis. Even though it is highly debated as to which dog breeds have the greatest CMT susceptibility or prevalence, we hypothesized that a cluster of CMT in these pedigrees is indicative of a genetic predisposition. Previous attempts to study CMT genetics either focused on small cohorts of multiple breeds or English Springer Spaniels (ESS) [15]. Multiple studies have indicated that the ESS from Sweden is a high-risk breed; however, it is worth noting that dogs in Sweden are rarely spayed - a procedure known to greatly reduce the risk of CMT [16–18]. Nevertheless, studying ESSs has revealed apparent CMT-associated SNVs, including ones in *BRCA1* and *BRCA2*, but the causative alleles have yet to be identified [19–21]. To our knowledge, there have been no published reports of WGS to investigate CMT inherited-genetic risk. Furthermore, outside of ESS, there have been no breed-specific analyses. Considering that different WGS efforts in dogs have recently proven to be advantageous in elucidating genetic susceptibility to disease [22–27], differences in body types [28], as well as adaptions against parasites [29], we have compiled and processed WGS data to begin the exploration of breed-specific CMT-risk alleles and, in this initial report, to specifically reveal the coding variants detected in orthologs of the high-risk human breast cancer susceptibility genes*.*

## Materials and methods

### CMT sample acquisition

DNA samples from 82 purebred CMT-affected dogs, representing 32 different American Kennel Club (AKC) recognized breeds (Table 1), were obtained from the Canine Health Information Center (CHIC) DNA Repository, which is a part of the Orthopedic Foundation for Animals (OFA; https://www.ofa.org/about/dna-repository). Briefly, this repository stores canine DNA samples and corresponding genealogic and phenotypic information to facilitate genetics research. Dog owners submit either blood or buccal samples to the repository along with their pets’ health history. Ultimately, researchers request access to samples pertaining to a disease of interest along with any additional information submitted. An unfortunate limitation of this resource is the lack of collected data. Being reliant on the owner’s knowledge and willingness to share, along with a generic survey used for all collected samples/phenotypes, information such as CMT pathology/histology, age of onset, and spay/neuter status were not provided to the research team.

**Table 1:** The total number of DNA samples from CMT-affected dogs from CHIC repository

Breed	Dogs per Breed	Dogs Connected to a Common Ancestor
Akita	1	–
Alaskan Malamute	2	–
Australian Cattle Dog	2	2
Beauceron	1	–
Bichon Frise	2	–
Border Terrier	1	–
Bouvier des Flandres	1	–
Boxer	1	–
Bullmastiff	1	–
Chesapeake Bay Retriever	1	–
Collie	1	–
Dalmatian	3	3
Doberman Pinscher	3	3
French Bulldog	1	–
Golden Retriever	18	18
Gordon Setter	4	4
Great Pyrenees	1	–
Irish Setter	2	2
Keeshond	2	2
Kerry Blue Terrier	1	–
Kuvasz	1	–
Leonberger	1	–
Mastiff	1	–
Newfoundland	4	4
Parson Russell Terrier	1	–
Pembroke Welsh Corgi	2	2
Petit Basset Griffon des Vendeen	2	–
Schipperke	1	–
Siberian Husky	8	7
Standard Schnauzer	7	7
Welsh Springer Spaniel	5	5
**Total dogs**	**82**	**59**
**Total breeds**	**32**	**12**

Of the 82 acquired samples, both blood-extracted DNA and buccal swabs were obtained. DNA was purified from the provided buccal swabs using the QIAamp DNA Mini Kit (Cat No./ID: 51304). Of the 32 represented breeds, 15 had multiple samples per breed (Table 1); thus, pedigree analyses were performed to identify breed-specific common ancestors and determine the level of relationship. Specifically, a dog’s registration and breeding information were entered into online (and mainly breed-specific) databases to build pedigrees. From this, 12 different pedigrees were generated.

### Sequencing and bioinformatics

**Fig. 1:**
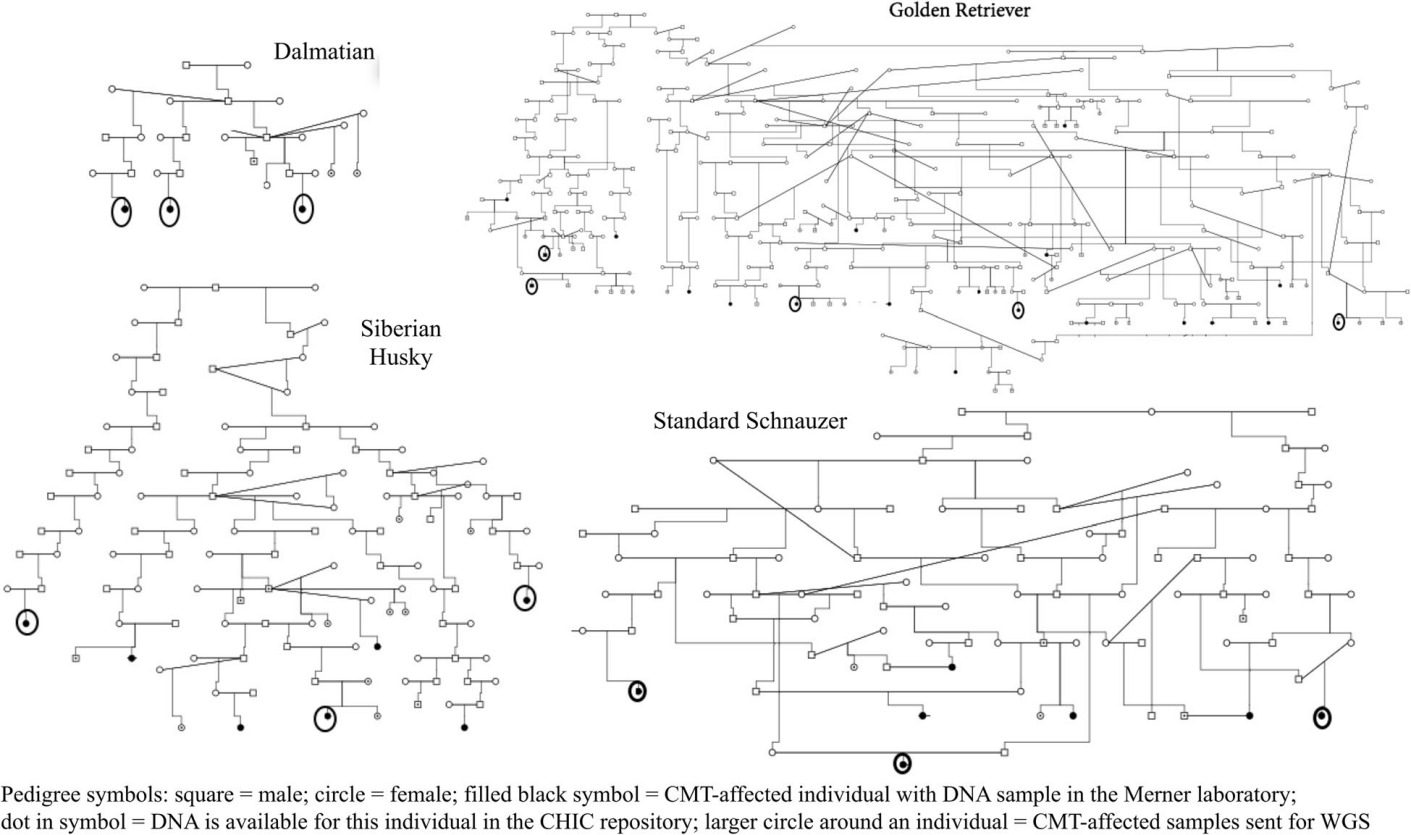
Purebred dog pedigrees and selected samples for WGS. Offspring of WGS samples are not depicted here, see Additional File 1 for offspring information

Fourteen DNA samples from four pedigrees were chosen for WGS. This included five Golden Retriever samples (three female, two male), three Siberian Husky females, three Standard Schnauzer females, and three Dalmatian females (Fig. 1). The selected dogs from each breed were AKC-registered and located within the same pedigree. Also, utilizing the CHIC database, offspring information of each dog was recorded to attempt to determine intact status as hormone exposure can affect the likelihood of development of CMT (Additional File 1).

**Fig. 2:**
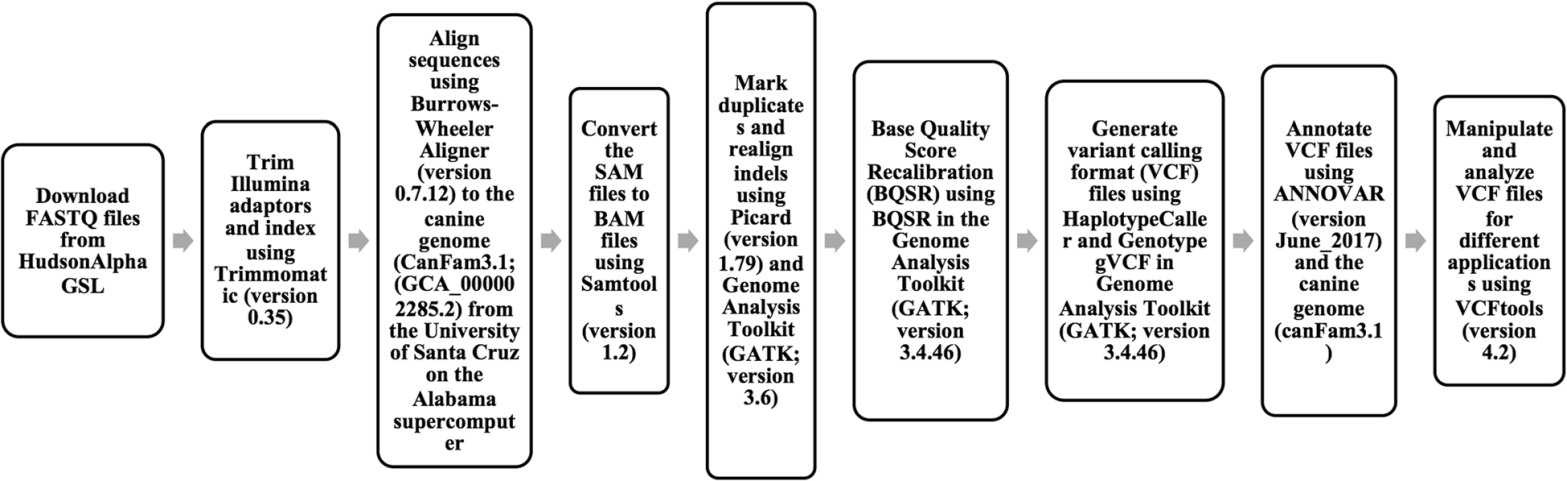
Bioinformatics pipeline for canine WGS data

Samples were prepared for Illumina platform WGS at HudsonAlpha Institute for Biotechnology’s Genome Sequencing Laboratory and the sequencing was carried out on Illumina HiSeq X. Paired FASTQ files were obtained from HudsonAlpha with sequencing data for each sample; the quality of the raw FASTQ files was determined using FASTQC. After assuring quality files, this sequencing data was carried through an in-house bioinformatics canine pipeline that was adapted from the Genome Analysis Toolkit (GATK) best practices bioinformatics pipeline [30] (Fig. 2). In brief, each sample file had Illumina adapters trimmed using the program Trimmomatic [31]. Samples were then aligned to the canine genome CanFam3.1 [32] using BWA mem [33]. Duplicate reads were marked using a Picard tool from version 1.79 (http://broadinstitute.github.io/picard/); then indels were realigned and base quality scores were recalibrated referencing the CanFam3.1 dbsnp data using Base Quality Score Recalibrator (BQSR) as part of the GATK v.3.4.46 [34]. Additionally, using GATK, coverage was calculated using the Depth of Coverage tool, and genomic variant calling format (GVCF) files were generated using Haplotype Caller and then merged through genotyping GVCF files. ANNOVAR [35] was used to annotate the VCF files using gene prediction from Ensembl build version 75. Variants were filtered by a Quality by Depth threshold of at least 12.

Coding variants within orthologs of human breast cancer susceptibility genes were isolated using the following coordinates: *BRCA1 (*ENSCAFT00000023190.4*)*: chr9:19960910–20,024,390, *BRCA2 (*ENSCAFT00000010309.3): chr25:7734450–7,797,815, *CDH1 *(ENSCAFT00000032333.3): chr5:80759112–80,834,940, *PTEN* (ENSCAFT00000024821.3): chr26:37853135–37,913,097, *STK11 *(ENSCAFT00000031055.3): chr20:57556289–57,625,288, and *TP53 *(ENSCAFT00000026465.3): chr5:32560598–32,574,109. All coding variants identified through WGS were validated through PCR and Sanger sequencing. Once the variant list was finalized, protein sequences for the orthologous human genes (*BRCA1* (NP_009231*)*, *BRCA2 (*NP_000050), *CDH1* (NP_004351), *PTEN* (NP_000305), *STK11* (NP_000446), and *TP53* (NP_000537)) were compared to the canine protein sequences (that corresponded to the above canine gene accession numbers) through EMBOSS Water alignment (https://www.ebi.ac.uk/Tools/psa/emboss_water/). These alignments were used to determine the corresponding human amino acid of each coding variant. The ClinVar database was then checked to see if a human mutation was identified in that position [36].

### Controls

Control data were obtained through Ensembl by accessing each canine gene’s variant table [37], which reports population genetic information from the European Variation Archive (EVA; https://www.ebi.ac.uk/eva/?eva-study=PRJEB24066). EVA provides data from the “High quality variant calls from multiple dog genome project – Run 1” representing WGS data of over 200 dogs from multiple breeds. Variants were filtered based on GATK’s best practices filtering guidelines, and the resulting variants and corresponding frequencies are accessible on the web through Ensembl’s database. Exact breed and sex information of these control dogs was unknown. This EVA control dataset is similar to the use of publically available databases that present general population control data for human disease genetic studies [38–42].

### Statistical analyses

For all the *BRCA1, BRCA2, CDH1, PTEN, STK11,* and *TP53* coding variants validated in the 14 CMT cases, allele frequencies were calculated in both cases and controls. Major and minor alleles were defined based on EVA control data. Subsequently, the Fisher Exact test was carried out to determine any statistically significant allele frequency differences between the EVA controls and the overall CMT cohort, as well as each specific breed. The Fisher Exact test, a test of contingency tables that calculates statistical significance based on a probability scale, is typically used as a statistical test for allele frequency [38, 43, 44]. This statistical analysis method has been considered a solution for analysis with small cell counts, which is why this analysis method was chosen for our analyses [45]. *P*-values were calculated using Fisher Exact test in R (v 3.5.1), which were not adjusted for multiple testing.

## Results

### Sequencing and annotation

WGS of the 14 dogs yielded an average sequencing depth of 26.0X (Table 2). On average, 99.13% of the reads aligned to the reference, resulting in 99.7, 99.1, 96.1 and 75.6% of the genome being covered at least 1X, 5X, 10X, and 20X, respectively (Table 2). An average of 7,909,896 variants were called for each dog, the majority of which were non-coding, with an average of 40,965 coding variants per dog. The overall average sequencing depth of the exome, according to Ensembl build version 75, was 25.6X; 99.8, 99.4, 96.7, and 76.0% of the exome was covered at least 1X, 5X, 10X, and 20X, respectively (Additional File 2).

**Table 2:** Whole genome coverage summary

Sample	Number of Mapped Reads to canFam3.1	% of Reads Mapped to canFam3.1	Average Sequencing Depth	% of bases covered greater than or equal to:								
				1X	5X	10X	15X	20X	25X	50X	75X	100X
Dal 1	432,798,423	99.0	29.0	99.7	99.3	98.9	97.8	92.3	73.6	1.2	0.5	0.3
Dal 2	479,265,395	99.1	24.6	99.7	99.3	98.7	95.3	79.0	45.9	0.8	0.4	0.2
Dal 3	517,919,216	99.1	27.4	99.7	99.3	98.8	97.3	89.1	64.7	1.0	0.4	0.3
Golden R 1	514,850,463	99.2	29.0	99.7	99.3	98.9	97.8	92.4	73.3	1.2	0.5	0.3
Golden R 2	521,394,202	99.3	29.2	99.7	99.3	98.9	97.8	92.7	74.5	1.2	0.5	0.3
Golden R 3	469,958,383	99.1	26.9	99.7	99.3	98.1	94.5	85.4	62.1	1.0	0.4	0.3
Golden R 4	420,815,898	99.0	23.4	99.6	99.1	97.2	91.0	72.4	39.3	0.7	0.3	0.2
Golden R 5	435,936,648	99.2	24.3	99.7	99.3	98.5	94.6	77.1	43.9	0.8	0.4	0.2
Sib H 1	439,440,441	99.2	25.1	99.7	99.2	98.6	95.7	81.3	49.3	0.9	0.4	0.2
Sib H 2	676,505,498	99.2	35.1	99.7	99.3	99.0	98.5	97.3	92.3	2.9	0.7	0.4
Sib H 3	306,005,622	99.2	16.0	99.6	98.9	91.7	57.2	18.0	3.6	0.4	0.2	0.1
Stand Sch 1	233,378,490	99.3	12.1	99.6	97.3	71.0	23.6	3.9	1.0	0.2	0.1	0.1
Stand Sch 2	716,837,887	98.7	36.6	99.7	99.5	99.2	98.7	97.6	93.5	4.2	0.8	0.5
Stand Sch 3	444,186,080	99.1	25.0	99.7	99.3	98.6	95.3	80.2	48.3	0.9	0.4	0.2
**Average**	472,092,332	99.1	26.0	99.7	99.1	96.1	88.2	75.6	54.7	1.2	0.4	0.3

### Variant analyses

A total of 19 coding variants, 13 nonsynonymous and six synonymous, were detected in *BRCA1*, *BRCA2*, *CDH1*, *PTEN*, *STK11,* and *TP53* (Table 3; Additional File 3). The nonsynonymous variants included ten missense variants (only one of which was considered possibly damaging based on Polyphen analysis), two non-frameshifting deletions, and one non-frameshifting indel (Table 3; Additional File 3). Of the 19 total variants, 11 had been previously reported in CMT canine cohorts (Table 3). Three *STK11* missense variants were identified (Table 3), one of which was detected in a single breed (Additional File 3). These three *STK11* variants have yet to be reported, not only in CMT studies, but also in the EVA control dataset (Table 3). Consequently, they appear to be associated with an increased risk of CMT, and each variant may affect breeds differently (Tables 3 and 4). Additionally, significant *P*-values were generated for *BRCA2* variants (Tables 3 and 4). Variants in other genes were noted but did not appear to be associated with disease.

**Table 3:** Summary of canine coding variants found within orthologs of human breast cancer susceptibility genes

Gene	RS ID Number	Variant Name	Protein Name	Variant Type	Polyphen Score	MAF in EVA Control Cohort (%)	MAF in CMT cases Cohort (%)	***P***-values (Total CMT Cases versus EVA Controls)	Initially Reported - CMT Heritability Study (Reference #)
BRCA1: ENSCAFT00000043953.1	rs397509570**	c.G3075A**	p.S1025S**	synonymous	NA	49.3	46.4	0.8465	*Borge* et al. 2011 [46]
BRCA2: ENSCAFT00000010309.3	rs23250374	c.A428G	p.H143R	missense	BENIGN	25.7	42.9	0.0749	*Yoshikawa* et al. 2008 [47]
	rs850935038**	c.T1158G**	p.C386W**	missense	BENIGN	20.6	42.9	**0.0095**	*Yoshikawa* et al. 2008 [47]
	rs851104585**	c.C2144A**	p.P715Q**	missense	BENIGN	0	0	1	–
	rs852009320**	c.C2154A**	p.S718S**	synonymous	NA	0	0	1	–
	rs851813778**	c.C2183T**	p.A728V**	missense	BENIGN	0	0	1	–
	rs851048998**	c.A2222G**	p.N741S**	missense	BENIGN	0	0	1	–
	rs23244160	c.A2401C	p.K801Q	missense	POSSIBLY DAMAGING	31.2	14.3	0.0868	*Borge* et al. 2011 [46]
	rs8676219	c.A4304G	p.K1435R	missense	BENIGN	25.9	42.9	0.0758	*Yoshikawa* et al. 2008 [47]
	rs397511123	c.6918_6920delGTT	p.L2307del	In frame deletion	NA	31.2	14.3	0.0868	*Borge* et al. 2011 [46]
	rs23255542	c.C6930T	p.F2310F	synonymous	NA	28.9	42.9	0.1359	*Yoshikawa* et al. 2008 [47]
	rs853007536**	c.9995_9996insAAA**	p.M3332delinsIK**	indel	NA	20.9	42.9	**0.0162**	*Yoshikawa* et al. 2005 [48]
CDH1: ENSCAFT00000032333.3	rs852509306	c.387_389delCCA	p.129delH	In frame deletion	NA	18.9	17.9	1	*Borge* et al. 2011 [46]
	rs397512866	c.C945T	p.S315S	synonymous	NA	12.3	14.3	0.7659	*Borge* et al. 2011 [46]
	rs851557759	c.A2448G	p.E816E	synonymous	NA	8.6	3.6	0.7187	–
PTEN: ENSCAFT00000024821.3	rs397513087	c.C909T	p.L303L	synonymous	NA	3.7	7.1	0.2970	*Borge* et al. 2011 [46]
STK11: ENSCAFT00000031055.3	–	c.C109T^	p.P37S^	missense	UNKNOWN	0	3.6	0.0654	–
	–	c.A286G^	p.M96V^	missense	BENIGN	0	10.7	**0.0003**	–
	–	c.T293C^	p.F98S^	missense	BENIGN	0	10.7	**0.0003**	–
TP53: ENSCAFT00000026465.3	no mutations were found								

** Major allele corresponds to the alternate allele, not the reference allele (based on EVA control data)

^ *P*-values for these variants were generated following the assumption that 200 of the control dogs were successfully sequenced in this location, and no mutations were identified

**Table 4:** Significant breed-specific *P*-values for nonsynonmous variants

Gene	RS ID Number	Variant Name	Protein Name	Variant Type	Polyphen Score	MAF in EVA Control Cohort (%)	CMT Cohort									
							**Total Cohort**		**Breed Specific**							
									**Dalmatian**		**Golden Retriever**		**Siberian Husky**		**Standard Schnauzer**	
							**MAF (%)**	***P*-value**	**MAF (%)**	***P*-value**	**MAF (%)**	***P*-value**	**MAF (%)**	***P*-value**	**MAF (%)**	***P*-value**
BRCA2: ENSCAFT00000010309.3	rs23250374	c.A428G	p.H143R	missense	BENIGN	25.7	42.9	0.0749	33.3	0.6506	70	**0.0048**	0	0.3447	50	0.1847
	rs850935038**	c.T1158G**	p.C386W**	missense	BENIGN	20.6	42.9	**0.0095**	50	0.1107	10	0.6948	83.3	**0.0096**	50	0.1107
	rs853007536**	c.9995_9996insAAA**	p.M3332delinsIK**	indel	NA	20.9	42.9	**0.01621**	50	0.1136	10	0.695	83.3	**0.0006**	50	0.1136
STK11: ENSCAFT00000031055.3	–	c.C109T^	p.P37S^	missense	UNKNOWN	0	3.6	0.0654	16.7	**0.0148**	0	1	0	1	0	1
	–	c.A286G^	p.M96V^	missense	BENIGN	0	10.7	**0.0003**	0	1	0	1	33.3	**0.0002**	16.7	**0.0148**
	–	c.T293C^	p.F98S^	missense	BENIGN	0	10.7	**0.0003**	0	1	0	1	33.3	**0.0002**	16.7	**0.0148**

** Major allele corresponds to the alternate allele, not the reference allele (based on EVA control data)

^ *P*-values for these variants were generated following the assumption that 200 of the control dogs were successfully sequenced in this location, and no mutations were identified

## Discussion

In an effort to study CMT heritability, our group acquired germline DNA from CMT-affected purebred dogs whose samples were submitted to the CHIC repository by the owner. Based on the hypothesis that dogs from the same breed/lineage share ancestral CMT-genetic risk factors, WGS was carried out on 14 samples from four generated pedigrees, including Golden Retriever, Siberian Husky, Standard Schnauzer, and Dalmatian. However, it is important to note that even if our hypothesis holds true in future studies that validate our findings or through novel CMT-gene discovery efforts, some cases within each pedigree could be phenocopies, representing sporadic cases not due to a familial genetic variant. This has to be kept in mind since ages of onset were not available through CHIC, and early ages of onset are associated with hereditary risk.

Our CMT-affected cohort represents dogs from the United States and did not include any ESS, which is the only breed to date that has had breed-specific CMT-genetic analyses [19–21]. To our knowledge, there have been no published reports of WGS to investigate the inherited risk of CMT. However, a compilation of next-generation sequencing efforts was used to compare human breast tumors to CMTs, and somatic mutations were identified [18]. Additionally, a limited number of studies have investigated germline CMT risk, and only a few risk variants have been identified with significance [15]. On our initial quest to find inherited breed-specific CMT-risk alleles, it is important to note that all CMT-affected dogs chosen for WGS were female except two closely related Golden Retrievers males. In addition to family history and early age of onset, male breast cancer is a hallmark of hereditary breast cancer in humans [11]; in fact, genetic predisposition significantly elevates the risk of male breast cancer, which is otherwise rare in the general population [49]. Therefore, assuming CMT genetic risk is similar to human genetic susceptibility, these two CMT-affected males suggest genetic factors are playing a role and were selected to enhance the prospects of discovery.

Unlike human disease gene discovery efforts, which have capitalized on whole-exome sequencing (WES) to facilitate discovery upon the introduction of next-generation sequencing [50], WGS has been the methodology of choice for identifying the genetic factors associated with inherited canine diseases. WGS and WES involve the re-sequencing of a genome or exome, respectively, which was made possible for canines once the first reference genome was published in 2005 [51]. In 2013, the first WGS [52–54] and WES [55] studies identified mutations associated with inherited canine disorders. Since that time, despite improvements to canine exome designs [56], the use of WES lagged behind. A possible reason for this is the cost. From our experience, when determining which of the two sequencing approaches to take for this study, the cost of WES was surprisingly expensive. WES baits alone were ~ $1000 per sample, which was the total cost per sample for WGS (to yield an average sequencing depth of at least 15X). Additional benefits to WGS include, (a) avoiding technical enrichment biases associated with WES capture, (b) more uniformity regarding sequencing-quality parameters, (c) the ability to explore both coding and non-coding regions, (d) the ability to better detect variants in coding regions (including regions targeted by a WES kit), and (e) the continued usefulness of the data as the annotation of the canine reference genome improves and gaps are filled [22, 32, 57–59].

Upon WGS of the 14 CMT-affected dogs, individual average sequencing depths ranged from 12.1 to 36.6X and overall averaged 26.0X. Aiming to achieve an average sequencing depth of, at least, 15X, all but one dog yielded such results (Table 2). Ultimately, the overall average was comparable to other canine WGS studies using Illumina technology [23–26]. On average, 99.7% of the genome was covered at least 1X, which is comparable to the Illumina-generated data in Gilliam et al. [23]. Noteworthy, it was higher than Sayyab et al. who used Ion Proton technology and reported an average sequencing depth of 9.2X and that 96% of the genome was covered at least 1X [22]. Viluma et al., who carried out another Ion Proton study, determined that 80% of the genome was covered at least 4X [60]; this is vastly different from our data, which covered 99.1% of the genome at 5X or greater. Similar to the two Ion Proton studies, our group also sought to determine the coverage of the canine exome through our WGS efforts. Not only did our study produce greater coverage for the canine genome, we additionally determined higher coverage results for the canine exome. Previously, Sayyab et al. reported that 91% of the exome was covered at least 1X, and Viluma et al. reported 77% of the exome was covered at least 4X. Contrarily, we obtained 99.8 and 99.4% of the exome at 1X and 5X, respectively (Additional File 2). In fact, these results far surpass the 5X coverage noted by Broeckx et al. regarding their improved canine exome design; they stated that just over 90% of the targeted base pairs were covered at least 5X [56]. Furthermore, Broeckx et al. had an average sequencing depth of 68.3X, which emphasizes the issue of lack of uniformity regarding targeted captures.

On average, each of the 14 dogs had 7.9 million variants called. Overall, this is comparable to the number of variants reported in the WGS studies that had similar sequencing depths [23–26]. The majority of the variants were non-coding, which, in the future, provides data for exploration. However, for this study, we focused on coding variants, specifically in orthologs of high-risk human breast cancer susceptibility genes, *BRCA1, BRCA2, CDH1, PTEN, STK11, and TP53* [11], as an initial gene exclusion approach, acknowledging that this dataset will be subsequently analyzed to investigate risk in other coding and non-coding regions of the genome. Through our initial analysis, 19 different coding variants were identified through WGS and confirmed through PCR and Sanger sequencing (Table 3). Interestingly, this list of variants gave insight regarding the complications of next-generation sequencing in dogs. Using a reference sequence derived from a Boxer for the alignment and, similarly, gene transcripts derived from the latest genome assembly for the annotation, we noted instances when the data could have easily been misconstrued. Firstly, four *BRCA2* variants were homozygous in all 14 CMT-affected dogs. This observation hinted that each alternate allele could in fact be the true wild-type (major) allele for the species since the four reference alleles appear to be unique to the Boxer. This was confirmed when we determined that all EVA control dogs were also homozygous for the four alternate alleles, as well as when we compared the Boxer reference protein sequence to the BRCA2 protein sequence for the Basenji (Basenji-breed-1.1) and the dingo dog (ASM325472v1). The reference genome is of an unaffected female Boxer, but that is the difficulty when studying a disease with age-related risk. These four *BRCA2* variants, with alleles that appear to be extremely rare in the species according to the control data, need to be further investigated to determine if they contribute toward disease risk in the Boxer. Unfortunately, we did not sequence any Boxers in this study, but their assessment would require a careful analysis of controls to properly interpret the data, which stresses that analyzing controls from multiple breeds can have extreme benefits.

**Fig. 3:**
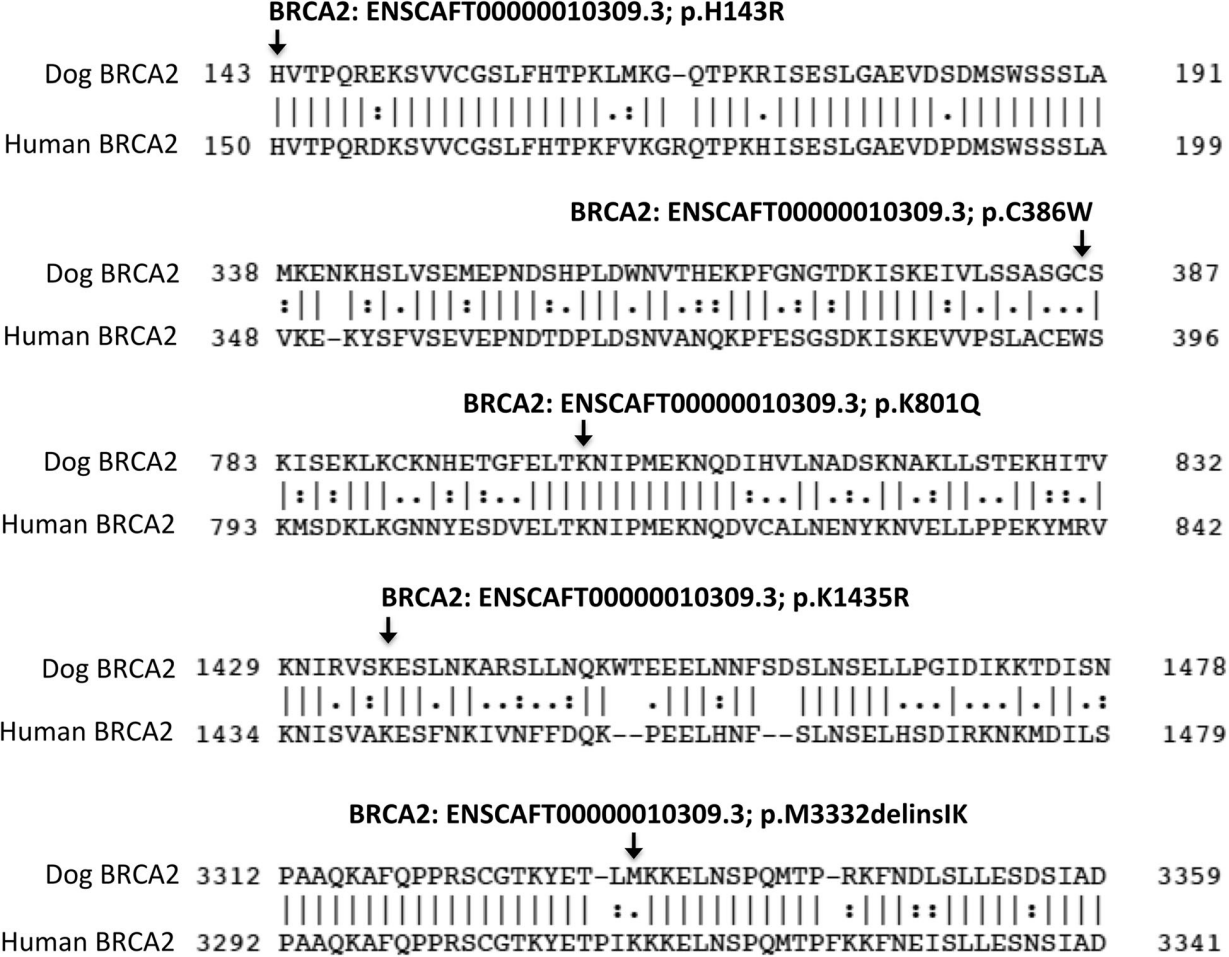
BRCA2 dog and human protein alignment for non-synonymous variants previously reported in CMT heritability studies

Similar to the example above, there were other instances where the alternate allele in the Boxer was in fact the major allele in controls. This was the case for two *BRCA2* variants that appear to be associated with CMT risk, particularly in the Siberian Huskies. According to the Boxer reference sequence and annotation using transcript ID ENSCAFT00000010309.3, these two variants were named *BRCA2*:c.T1158G (p.C386W) and *BRCA2*:c.9995_9996insAAA (p.M3332delinsIK), which were previously reported in CMT heritability studies [46–48, 61]. Thus, the Boxer had a cysteine at amino acid 386 and a methionine at 3332. However, the major allele in the EVA control dogs translated to most dogs having a tryptophan at amino acid 386, and isoleucine-lysine at position 3332, which also resembles that of the references for Basenji dog breed and the dingo dog and, most interestingly, corresponds to the conserved human residues. Comparing allele frequencies between the CMT cases and EVA controls revealed that cysteine at amino acid 386 and a methionine at 3332 were associated with an increased risk of CMT. In fact, these alleles appear to be most strongly associated with CMT risk in Siberian Huskies (Table 4). These associations will need to be validated by studying larger cohorts. Boxers should also be studied to determine the true allele frequencies in that breed. If a cysteine at position 386 and a methionine at 3332 are actually more common in Boxers, they could be at an elevated disease risk. Noteworthy, the human BRCA2 residue W395 corresponds to W386 in these dogs (Fig. 3), and while a cysteine mutation at W395 has not been found in human hereditary breast cancer cases, two pathogenic truncation mutations have been reported at that position (ClinVar Variation IDs: 266612 and 265053), along with the missense variant, W395G, which is considered a variant of unknown significance (VUS; ClinVar Variation ID: 51078) [36]. Similarly, human BRCA2 residues I3312 and K3313 correspond to the conserved isoleucine-lysine in dogs at 3332 (Fig. 3), and BRCA2 p.I3312M has been reported as another VUS (ClinVar Variation ID: 52921) [36]. VUS are genetic variants of unknown clinical significance, and it has been reported that as many as 15% of people who undergo *BRCA1* and *BRCA2* genetic screening are informed of a detected VUS, which are inconclusive results [62].

In addition to *BRCA2* c.T1158G (p.C386W), we identified three other *BRCA2* missense variants that had been previously reported in CMT studies assessing inherited risk; this included *BRCA2*:c.A428G (p.H143R), *BRCA2*:c.A2401C (p.K801Q), and *BRCA2*:c.A4304G (p.K1435R; Table 3) [15, 46, 47]. Even though neither of these variants generated a significant *P*-value when investigating the overall CMT cohort, those *P*-values appeared to be trending towards significance. Nonetheless, breed-specific analyses suggested that BRCA2 p.H143R is associated with CMT-risk in Golden Retrievers (Table 4). This variant was previously described as possibly damaging by Borge et al. [46, 63], but PolyPhen2 analysis predicts it to be benign [64]. Similarly, contradictory pathogenicity predictions were noted for BRCA2 p.K801Q. It was predicted to be possibly damaging using PolyPhen2 but was initially reported by Borge et al. in 2011 and predicted to be benign [46, 63]. Moreover, the Polyphen2-suggested benign variant, p.K1435R, was reported by Yoshikawa et al. in 2008 as possibly deleterious upon blood and CMT analyses, including loss-of-heterozygosity studies [47]. Altogether, knowing that current computational prediction methods misclassify a significant percentage of clinically valid missense variants [65], and that the P-values generated for those variants were, at least, trending towards significance, larger genotyping and functional studies will be required for true classification. Additionally, all three missense variants are conserved in humans (Fig. 3), and, most interestingly, the equivalent mutations of canine p.H143R and p.K1435R have been identified in humans as p.H150R and p. K1440R, respectively (ClinVar Variation IDs: 51657 and 51632) [36]. These variants are classified as VUS, similar to the other *BRCA2* VUS mentioned above. Overall, VUS include missense variants as well as in-frame insertions and deletions, both of which were detected in this study; this overlap with human and dogs offers another avenue for exploration since the reclassification of VUS is a current hot topic [66, 67].

Regarding the other assessed genes, *STK11* displayed the most interesting results. Three missense variants were identified, *STK11* c.C109T (p.P37S), *STK11* c.A286G (p.M96V), and *STK11* c.T293C (p.F98S), all of which appear to play a role in CMT risk. Our findings suggest that *STK11* is a CMT susceptibility gene, corroborating a similar claim in a recent publication by Canadas et al. [68]. Canadas and colleagues suggested that the minor allele (T) of rs22928814, which lies within an intron of *STK11,* was associated with an increased risk of CMT. Interestingly, this allele, which the authors reported to have a frequency of 25.7 and 14.9% in cases and controls, respectively [68], has a frequency of 26.6% in EVA controls according to Ensembl [37], which is more similar to the frequency reported in the CMT cases and stresses the need for validation studies. Of note, this variant was not detected in any of the CMT-affected dogs sequenced in this study. However, the three missense variants identified in this study appear to be extremely rare alleles since they were not reported in EVA controls. Regarding STK11 p.M96V and p.F98S, breed-specific *P*-values of 0.0002 and 0.0148 were generated for the Siberian Huskies and Standard Schnauzers, respectively (Table 4). Additionally, STK11 p.P37S was only detected in one Dalmatian, and breed-specific analyses suggest that this variant possibly increases risk of CMT in that breed. Overall, these findings mimic the phenomena in humans that rare *STK11* variants increase risk of disease [11]. However, it is worth noting that these variants are not in a conserved region with human STK11 protein sequence. How these *STK11* variants, along with the identified *BRCA2* variants, specifically contribute towards risk needs to be further studied. Firstly, variants in both *STK11* and *BRCA2* appear to be tightly linked, thus determining the true risk alleles in both *BRCA2* and *STK11* is important. Also, polygenic risk assessment in humans is another hot topic [69], and demonstrating the same concept in dogs would further validate their usefulness as a model of hereditary breast cancer [15].

## Conclusions

To our knowledge, we carried out the first study to assess inherited CMT risk through WGS data analysis, and we investigated risk through both multiple breed and breed-specific analyses. This manuscript reports the variants detected in six orthologs of high-risk human breast cancer susceptibility genes as an initial gene exclusion approach, acknowledging that this WGS dataset will be subsequently analyzed to investigate risk in other coding and non-coding regions of the genome. Through our initial efforts, we identified variants in *BRCA2* and *STK11* that appear to be associated with CMT risk. These variants could alter risk in many breeds but appear to be more prevalent in some breeds compared to others. Additionally, we identified several *BRCA2* variants that correspond to VUS in humans. Indeed, these results need to be validated; the identified variants now require further investigation to determine the role they play in risk in both humans and dogs, which we plan to promptly address. For instance, noting the limitation of using a control dataset of multiple unknown breeds, we plan to acquire control samples to determine breed-specific allele frequencies. Furthermore, functional studies are pertinent to determine pathogenicity. Ultimately, in addition to this initial gene exclusion effort, this dataset provides the opportunity for novel discovery and has the potential to lead to further breakthroughs in canine and human breast cancer research through comparative analyses. Overall, in the era of personalized medicine, identifying risk variants not only provides better risk assessment and opportunities to selectively breed out a pathogenic mutation, it also can provide insight towards disease mechanism and aid in the development of targeted therapies [10, 70].

## Supplementary information

**Additional file 1.** Table summary of offspring information from CHIC repository for the 14 WGS samples.

**Additional file 2.** Table of exome Coverage Summary for the 14 canines sequenced. Exome regions according to Ensembl build version 75 for CanFam3.1.

**Additional file 3.** Detailed summary of canine coding variants found within orthologs of human breast cancer susceptibility genes.

## Data Availability

The dataset supporting the conclusions of this article is available upon request through communication with the corresponding author. The DNA samples that were sequenced during this project are available through the CHIC repository (https://www.ofa.org/about/dna-repository). Sample selection can be shared through communication with the corresponding author.

## Abbreviations

RCND: Renal cystadenocarcinoma and nodular dermatofibrosis
*BHD*: *Birt-Hogg-Dubé*
CMT: Canine mammary tumor
WGS: Whole genome sequencing
ESS: English Springer Spaniels
AKC: American Kennel Club
CHIC: Canine Health Information Center
OFA: Orthopedic Foundation for Animals
GATK: Genome Analysis Toolkit
GVCF: Genomic variant calling format
EVA: European Variation Archive
SNV: Single nucleotide variant
WES: Whole exome sequencing
VUS: Variants of unknown significance
